# Mapping the evidence on lifestyle diseases: protocol for a systematic scoping review

**DOI:** 10.1186/s13643-026-03196-9

**Published:** 2026-05-14

**Authors:** Marcin Golec, Hanna Mycka, Tomasz Wieczorek, Marek Tomaszewski, Julia Suchcicka, Cezary Dmowski, Patryk Banski, Kamil Kogut, Julia Janas, Julia Bogucka, Dominika Dachnij, Amelia Kolomanska, Katarzyna Matczyszyn, Sandra Barteit

**Affiliations:** 1 Heidelberg Institute of Global Health (HIGH), Faculty of Medicine and University Hospital, Heidelberg University, Heidelberg, Germany; 2 Interdisciplinary Society for Prevention of Lifestyle Diseases, Wroclaw, Poland; 3 Faculty of Chemistry, Wrocław University of Science and Technology, Wroclaw, Poland; 4 WPI-SKCM2, Hiroshima University, Hiroshima, Japan

**Keywords:** Lifestyle diseases, Scoping review, Civilizational diseases, Civilization diseases

## Abstract

**Background:**

Lifestyle diseases are commonly described as conditions arising from long-term behavioural patterns, occupational exposures, and broader civilisational and environmental determinants. Although the term is frequently used in public health and clinical literature, its conceptual boundaries remain heterogeneous and inconsistently defined. While extensive research exists on individual non-communicable diseases and lifestyle risk factors, no comprehensive scoping review has systematically mapped how “lifestyle diseases” are defined, operationalised, and studied across behavioural, occupational, environmental, and technological domains.

**Methods:**

This protocol outlines a systematic scoping review that aims to map the scientific literature on lifestyle diseases, identify which clinical conditions are labelled as such, examine underlying determinants and contributing factors, describe reported prevention strategies, and identify research gaps in the field. The review will be conducted following the methodological framework of Arksey and O’Malley, refined by Levac et al., and reported in accordance with the PRISMA-ScR guidelines. Five electronic databases (MEDLINE/PubMed, Scopus, CINAHL, Web of Science, and APA PsycInfo) will be searched for peer-reviewed literature published from 1990 to the date of search execution. Eligibility criteria will be defined using a structured framework and will include studies that explicitly address or conceptualise lifestyle diseases.

**Discussion:**

Findings will be synthesised descriptively and presented using thematic categorisation, tabular summaries, and graphical visualisations to map disease categories, determinants, preventive approaches, and geographic distribution. This review will provide a structured overview of how lifestyle diseases are defined and studied in the scientific literature. By clarifying conceptual boundaries and identifying research gaps, the review aims to support interdisciplinary research and inform future public health investigations.

**Supplementary Information:**

The online version contains supplementary material available at 10.1186/s13643-026-03196-9.

## Introduction—rationale, need, and aim of the study

### Background

#### Definition of lifestyle diseases

The term lifestyle diseases is commonly used to describe conditions that are attributed to the long-term impact of daily behaviours and living conditions. It typically refers to a heterogeneous group of chronic disorders, predominantly non-communicable, associated with persistent behavioural patterns and exposure to broader civilizational factors [1, 2]. The concept is frequently used interchangeably with the terms civilisational diseases or civilisation diseases [1].

Although closely related to non-communicable diseases (NCDs), the two terms are not synonymous. NCDs encompass all diseases that are not transmissible from person to person and include genetic, rare, orphan, and autoimmune conditions that are not primarily linked to lifestyle determinants. In contrast, lifestyle diseases are generally framed as conditions in which modifiable behavioural, environmental, or occupational exposures play a central role. Some literature further highlights intersections between behavioural, environmental, and infectious determinants (e.g. tuberculosis [3] or dental caries [4–6]), illustrating the conceptual ambiguity surrounding the term and its boundaries.

#### Drivers: underlying causes, determinants, and contributing factors

Lifestyle diseases are commonly associated with behavioural determinants, including sedentary lifestyle, tobacco use, alcohol consumption, and unhealthy dietary patterns [7]. Diets rich in simple sugars and saturated fats contribute to obesity and metabolic dysregulation, which in turn are linked to diabetes mellitus, cardiovascular diseases, hypertension, atherosclerosis, and dyslipidaemia [7, 8].

Beyond individual behaviour, occupational conditions represent another important domain of exposure. Work in traditional industries may involve contact with organic dust, for example in agriculture and food processing [9]. Physical workload may contribute to musculoskeletal disorders, while employment in heavy industry can entail exposure to asbestos or mineral and coal dust [10]. Office-based and corporate work environments are increasingly associated with psychological stressors and psychosomatic health outcomes [11]. Contemporary workplace organization and management structures have likewise been discussed in relation to employee well-being and mental health [12–14].

The broader socio-economic context further shapes lifestyle patterns and living standards and may contribute to the development and progression of lifestyle-related conditions [15, 16]. Socio-economic inequalities influence access to healthy environments, education, nutrition, and healthcare, thereby affecting risk exposure and health outcomes. Psychosocial factors, including lack of meaning (purpose) in life, alienation, and loss of culture and/or identity, have been also proposed as important determinants of lifestyle diseases [17].

Technological developments have introduced additional dimensions to the discourse. The widespread use of digital devices in both occupational and leisure contexts has been associated with health effects linked to screen time [18]. Emerging discussions also address potential impacts of artificial intelligence and digital technologies on mental and behavioural health [19, 20]. These developments reflect the evolving and expanding ways in which the concept of lifestyle diseases is used in contemporary literature.

Population ageing represents another dimension frequently discussed in relation to lifestyle-related chronic disease burden. Increased life expectancy and demographic shifts have contributed to a higher prevalence of chronic, age-associated conditions, sometimes described as a “diseasome of ageing”, including chronic kidney disease, type 2 diabetes, fatty liver disease, cardiovascular diseases, cancer, depression, osteoporosis, and dementia [8]. In addition, environmental and planetary determinants, such as climate-related exposures (e.g. heat stress) and pollution, are increasingly discussed in relation to chronic disease risk [8].

#### Burden of lifestyle diseases

The global burden of chronic disease has risen substantially over recent decades [21], reflecting profound changes in behavioural patterns, occupational environments, socio-economic structures, and technological development [7, 22]. According to the World Health Organization, non-communicable diseases account for approximately 74% of global deaths [8]. Many of these conditions are widely described in the literature as being strongly influenced by modifiable lifestyle and environmental determinants, underscoring their major public health relevance.

#### Research gap

Despite the widespread use of the term across disciplines and contexts, its conceptual scope remains inconsistently defined. Lifestyle diseases receive considerable scientific, clinical, societal, and political attention [20]. Numerous studies focus on specific conditions or individual behavioural risk factors. However, despite frequent use of the term in scientific discourse, there is a lack of comprehensive synthesis examining how “lifestyle diseases” are defined, which clinical conditions are included under this label, and how determinants and preventive strategies are conceptualized across behavioural, occupational, environmental, and technological domains.

Given the dynamic changes in living environments, work structures, digital technologies, and demographic trends, an updated and systematic mapping of the literature is warranted. To date and according to our best knowledge, no comprehensive scoping review has addressed the full spectrum of how lifestyle diseases are framed and studied across disciplines.

### Objectives

The objective of this study is to conduct a systematic scoping review to map and synthesise the scientific literature on lifestyle diseases. Specifically, the review aims to examine how the term “lifestyle diseases” is conceptualized and applied in the literature, to identify associated determinants and preventive approaches, and to explore gaps in current research.

To address this objective, the review will seek to answer the following research questions:

- Which clinical conditions are identified or labelled as “lifestyle diseases” in the scientific literature?
- What underlying causes, determinants, and contributing factors are associated with lifestyle diseases?
- What emerging behavioural, occupational, environmental, socio-economic, psychosocial, or technological factors are discussed in relation to evolving lifestyle-related conditions?
- What preventive measures or interventions are described in relation to lifestyle diseases?
- What research gaps or unmet needs are identified within the lifestyle diseases domain?

## Methods

A systematic scoping review was selected as the most appropriate approach to address the study objectives and to map the breadth and characteristics of existing literature in this field [23]. The review will be conducted in accordance with the five-stage methodological framework proposed by Arksey and O’Malley [24] and further refined by Levac et al. [25]. This protocol is reported in line with the Preferred Reporting Items for Systematic Review and Meta-Analysis Protocol (PRISMA-P) checklist, adapted as appropriate for a scoping review (items not applicable to a scoping review protocol are marked as not applicable in the completed checklist). The completed scoping review will be reported in accordance with the Preferred Reporting Items for Systematic Reviews and Meta-Analyses Extension for Scoping Reviews (PRISMA-ScR) [26].

### Information sources and search strategy

The literature search will include five electronic databases: MEDLINE (via PubMed), Scopus, CINAHL, Web of Science, and APA PsycInfo [27]. The search strategy was developed by members of the review team with sector-specific expertise in lifestyle diseases, public health, clinical psychology, and medicine. Search terms were identified iteratively based on the review objectives, preliminary scoping of the literature, terminology used in key publications, and team expertise. The initial MEDLINE/PubMed search strategy was developed first and then adapted for Scopus, CINAHL, Web of Science, and APA PsycInfo. No expert librarian or information specialist was involved in the development of the search strategy, and no formal PRESS assessment was conducted. However, the search strings were reviewed internally by the research team and refined through pilot searches to improve sensitivity and ensure that relevant terminology, including “lifestyle diseases”, “civilisational diseases”, and “civilization diseases”, was captured (see Additional file 1). Grey literature will be identified through a structured Google Scholar search [28] and, where relevant, manual searches of institutional reports (e.g. WHO, OECD, ILO) and reference lists of included studies. The search will cover literature published from January 1990 to the date of final search execution. The cut-off year of 1990 was selected to reflect contemporary conceptualizations and methodological approaches within the field.

### Eligibility criteria

Eligibility criteria will be developed using the PCC framework (Population, Concept, Context)—see Table 1 for further details [29]. Studies will be eligible for inclusion if they explicitly address, define, conceptualise, or label one or more conditions as “lifestyle diseases” or related terms (e.g. civilizational diseases). Both empirical and conceptual studies published in peer-reviewed journals indexed in internationally recognised databases (e.g. PubMed/MEDLINE, Scopus, APA PsycInfo) will be considered. Grey literature from recognised institutional sources will also be eligible. Publications in languages other than English will be excluded due to feasibility constraints. While this may introduce language bias, previous research suggests that its overall impact on findings is likely limited [30]. Editorials, letters to the editor, commentaries, press articles, and records without accessible full texts will be excluded. As this is a scoping review aimed at mapping conceptualisation and research characteristics, no formal critical appraisal of included studies will be conducted.

**Table 1:** PCC (Population, Concept, Context) framework

Population	All human populations (no restrictions on age, sex, or location)
Concept	Sources that explicitly address, define, conceptualize, operationalize, classify, or label one or more conditions as “lifestyle diseases” or closely related terms, including “civilisational diseases” or “civilization diseases”. Sources may discuss associated clinical conditions, determinants, contributing factors, prevention strategies, interventions, recommendations, or research gaps
Context	Any geographical, socio-economic, cultural, occupational, healthcare, environmental, technological, or community context. No restrictions will be applied by country, region, income setting, healthcare system, or setting of care English language only

### Study selection

All records identified through database searches will be imported into a structured screening form developed in Microsoft Excel (see Additional files 2a and 2b). After removal of duplicates, titles and abstracts will be screened independently by two reviewers. Prior to full screening, a calibration exercise will be conducted on a subset of records to ensure consistent interpretation of eligibility criteria. Full texts of potentially eligible studies will then be assessed independently by two reviewers to confirm final inclusion. Discrepancies between reviewers will be resolved through discussion; if necessary, a third reviewer will adjudicate.

### Data extraction

A standardised data-charting form will be used to extract and map relevant information from included studies (see Additional file 2c). The form was developed to capture information aligned with the review objectives and research questions. Data extraction will be conducted independently by two reviewers.

Extracted variables will include:

- Study characteristics (study design, sample size, population age group, gender distribution)
- Conceptualisation of lifestyle diseases (definitions used, terminology applied)
- Clinical conditions identified as lifestyle diseases
- Primary lifestyle-related determinants discussed (behavioural, occupational, socio-economic, psychosocial, environmental, technological)
- Preventive measures, interventions, or recommendations described
- Funding sources and reported conflicts of interest
- Research gaps, unmet needs, or future directions identified by the authors

To assess feasibility and consistency of the proposed approach, a pilot study involving ten papers was conducted (see Additional file 3). These pilot findings support the feasibility of the study protocol, in particular the key data charting form.

### Data synthesis and presentation

Findings will be synthesised descriptively and presented through thematic categorisation, tabular summaries, and graphical representations to map the scope and distribution of evidence.

Namely, the findings of the scoping review will be presented in multiple complementary formats to reflect the breadth and heterogeneity of the identified literature. Descriptive characteristics of included studies (e.g. study design, sample size, population characteristics) will be summarized in tabular form.

Results will be structured according to:

- Clinical conditions identified as lifestyle diseases
- Key lifestyle-related determinants (behavioural, occupational, socio-economic, psychosocial, environmental, technological)
- Socio-economic dimensions addressed
- Geographic distribution of studies

Findings will additionally be organised to align with the predefined research questions. This will include mapping how lifestyle diseases are conceptualised across disciplines, identifying underlying causes and contributing factors, and summarising reported preventive strategies and recommendations. Research gaps and unmet needs identified within the literature will also be systematically charted. In line with best practices for scoping reviews, results will be presented through descriptive summaries supported by tables, figures, and graphical visualisations, including geographic mapping where appropriate.

## Discussion

The proposed systematic scoping review aims to provide a structured and comprehensive mapping of how lifestyle diseases are conceptualised and studied in the scientific literature. By synthesising definitions, determinants, underlying causes, emerging trends, preventive measures, and identified research gaps, the review seeks to clarify the scope and application of the term “lifestyle diseases” across disciplines.

The term “lifestyle diseases” is widely used in scientific, clinical, and policy discourse, yet its boundaries and operationalisation vary considerably. Such conceptual heterogeneity may limit comparability across studies, complicate epidemiological surveillance, and influence how research priorities and public health strategies are framed. By systematically mapping definitions and applications of the term, this review aims to contribute to greater conceptual transparency and support more consistent use of terminology in future research.

Given the heterogeneity of conditions and determinants associated with this concept, the review is expected to serve as an interdisciplinary reference point for researchers and clinicians. By organising the available evidence in a systematic and transparent manner, the review will facilitate identification of thematic clusters, disciplinary perspectives, geographic patterns, and areas requiring further investigation. A scoping review methodology was selected because the objective is to explore the breadth and diversity of conceptualisations rather than to assess intervention effectiveness or generate pooled quantitative estimates. The anticipated variation in study designs, disciplinary approaches, and definitions makes a scoping framework more appropriate than a traditional systematic review. The focus on breadth enables comprehensive mapping, while acknowledging that analytical depth and formal quality appraisal are beyond the scope of this methodology.

Several limitations are anticipated. As inherent to scoping review methodology, the emphasis lies on capturing a broad overview rather than conducting in-depth critical evaluation. No formal quality appraisal of included studies will be performed, which may limit conclusions regarding strength of evidence. The restriction to English-language publications may introduce language bias, and reliance on selected databases may contribute to indexing bias. Furthermore, publication patterns may lead to underrepresentation of research from low- and middle-income settings or non-English-speaking regions. Although inclusion of grey literature from recognized institutions may mitigate some of these limitations, they cannot be fully eliminated.

Overall, the proposed review aims to provide a transparent and structured foundation for future research on lifestyle diseases by clarifying conceptual boundaries, mapping determinants and preventive approaches, and identifying underexplored domains within this evolving field.

## Conclusions

This scoping review will provide a structured overview of how lifestyle diseases are defined, conceptualised, and studied in the scientific literature. By systematically mapping associated conditions, determinants, preventive strategies, and identified research gaps, the review will clarify the scope and application of the term across disciplines. The findings are expected to support future research by identifying areas of conceptual ambiguity, thematic concentration, and underexplored domains within the lifestyle diseases field.

## Supplementary Information

Additional file 1.

Additional file 2.

Additional file 3.

## Data Availability

All data generated and analysed during this study are included in the article and its supplementary information files.

## Abbreviations

APA PsycInfo: American Psychological Association PsycInfo Database
CINAHL: Cumulative Index to Nursing and Allied Health Literature
ILO: International Labour Organization
MEDLINE: Medical Literature Analysis and Retrieval System Online
NCDs: Non-communicable diseases
OECD: Organisation for Economic Co-operation and Development
PCC: Population, Concept, Context
PRISMA-ScR: Preferred Reporting Items for Systematic Reviews and Meta-Analyses Extension for Scoping Reviews
WHO: World Health Organization
