# Supplementary material for: Mapping the evidence on lifestyle diseases: protocol for a systematic scoping review
Source: Syst Rev. 2026 May 14;15:167. doi: 10.1186/s13643-026-03196-9 (PMC13173829; doi:10.1186/s13643-026-03196-9)
Supplement: Supplementary file 1 — Additional file 1. [file 13643_2026_3196_MOESM1_ESM.docx]

**Additional file 1**. Databases search terms.

| **Database** | **Search string** |
| --- | --- |
| Medline | ("lifestyle disease" OR "lifestyle diseases") OR ("life style disease" OR "life style diseases") OR ("civilization disease" OR "civilization diseases") OR ("civilisation disease" OR "civilisation diseases") |
| Scopus | ("lifestyle disease" OR "lifestyle diseases") OR ("life style disease" OR "life style diseases") OR ("civilization disease" OR "civilization diseases") OR ("civilisation disease" OR "civilisation diseases") |
| APA PsycInfo | ("lifestyle disease" OR "lifestyle diseases") OR ("life style disease" OR "life style diseases") OR ("civilization disease" OR "civilization diseases") OR ("civilisation disease" OR "civilisation diseases") |
| CINAHL | ("lifestyle disease" OR "lifestyle diseases") OR ("life style disease" OR "life style diseases") OR ("civilization disease" OR "civilization diseases") OR ("civilisation disease" OR "civilisation diseases") |
| Web of Science | ("lifestyle disease" OR "lifestyle diseases") OR ("life style disease" OR "life style diseases") OR ("civilization disease" OR "civilization diseases") OR ("civilisation disease" OR "civilisation diseases") |

Using the SCOPUS platform, only titles were searched for all search terms. For Medline, the text word search was prioritized to broaden the coverage for the emerging topic of diseases resulting from exposure to digital technologies.
