# Supplementary material for: Mapping the evidence on lifestyle diseases: protocol for a systematic scoping review
Source: Syst Rev. 2026 May 14;15:167. doi: 10.1186/s13643-026-03196-9 (PMC13173829; doi:10.1186/s13643-026-03196-9)
Supplement: Supplementary file 2 — Additional file 2. [file 13643_2026_3196_MOESM2_ESM.pdf]

Additional file 2a. Relevance screening form on basis of bibliographic data (title and abstract).

	Ref No:						
	Authors:						
	Title:						
	Year of publication, DOI/PMID, journal and other bibliographic data:						
**No**	**Question**	**Answer**	**Answer's options**	**Exclusion criteria**	**Decision**	**Decision's options**	**Additional notes**
1	What type of source is the reference?		Peer reviewed journal article (1); Conference Paper (2); Book Section (3); Unpublished Work (4); Commentary (5); Editorial (6); Systematic review (7); Thesis (8); Protocol (9); Report (10); Grey literature (11); Other e.g., press article (12)	Result of category “others”		Inclusion (1); Exclusion (0); Unclear (2)	
2	Is an abstract available? (If no abstract, is the full text available?)		Yes, abstract and/or full text is available (1), No (2)	No abstract available and full text not accessible		Yes (1); No (0); Unclear (2)	
3	From the title and abstract, it can be concluded that the article focuses on lifestyle/civilisation diseases.		Yes (1), No (2)	No		Yes (1); No (0); Unclear (2)	
	**Screening decision:**					Included (1); Excluded; Unclear (2)	

Additional file 2b. Eligibility Screening form on basis of full-text screening.

	Ref No:						
	Authors:						
	Title:						
	Year of publication, DOI/PMID, journal and other bibliographic data:						
**No**	**Question**	**Answer**	**Answer's options**	**Exclusion criteria**	**Decision**	**Decision's options**	**Additional notes**
1	Is the full text available?		Yes (1); No (2)	No		Included (1); Excluded; Unclear (2)	
2	Is the full text in English?		Yes (1); No (2)	No		Included (1); Excluded; Unclear (2)	
3	Again: What type of source is the result?		Peer reviewed journal article (1); Conference Paper (2); Book Section (3); Unpublished Work (4); Commentary (5); Editorial (6); Systematic review (7); Thesis (8); Protocol (9); Report (10); Grey literature (11); Other e.g., press article (12)	Publication type is categorized as "Other" (non-scholarly source)		Included (1); Excluded; Unclear (2)	
4	The article is focused on human health, it is not an animal model nor in vitro study.		Yes (1); No (2)	No		Included (1); Excluded; Unclear (2)	
5	The article addresses life style diseases.		Yes (1); No (2)			Included (1); Excluded; Unclear (2)	
	**Screening decision:**					Included (1); Excluded; Unclear (2)	

Additional file 2c. Characterization form on basis of full-text analysis.

	Ref No:			
	Authors:			
	Title:			
	Year of publication, DOI/PMID, journal and other bibliographic data:			
**No**	**Data Item**	**Description (to be extracted)**	**Response**	**Options / Examples**
1	**Study location**	Country or countries where the study was conducted.		free text (e.g., USA; India or unspecified) / M49 country code
2	**Study region**	Geographic region of the study setting (UN M49 classification)		region name as per UN M49 standard (https://unstats.un.org/unsd/methodology/m49/)
3	**Income level**	Country income level (World Bank classification).		low (1); lower-middle (2); upper-middle (3); high (4) or unspecified (0)
4	**Study design**	Study design or type.		clinical study - cross sectional (1); clinical study - cohort (2); randomized controlled trial (3); qualitative study (4); mixed-methods research (5); clinical study - other, explain in "additional notes" on the right (6); scoping review (7), systematic review (8), metaanalysis (9), review - other (10)
5	**Sample size**	Number of participants (or number of studies, if a review).		<100 / small (1); 100–500 / medium (2); >500 / large (3); unspecified / not applicable (0)
6	**Population age group**	Age group(s) of the study population.		children: 0–1 y.o. (1); adolescents 13–18 y.o. (2); adults: 19–64 y.o. (3); older adults: 65+ y.o. (4); general population, mixed or not age-specific (5), unspecified/other (0)
7	**Sex/gender**	Sex/gender composition of the study population.		male (1); female (2); both (3); unspecified (0)
8	**Lifestyle factor category**	Primary category of lifestyle factors addressed.		behavioral (1); occupational (2); technological (3); aging-related (4); environmental (5), other (specify) (6)
9	**Funding source**	Source of study funding.		public (govt/non-profit); private (industry); mixed; none, not reported
10	**Conflict of interest**	Reported conflicts of interest.		yes (1); no (0); not reported (2)
11	**Socio-economic factors**	Socio-economic factors considered (e.g., income, education); list any mentioned or “None” if not reported.		free text (e.g., income, education)
12	**Study aims**	Stated goals or objectives of the study (especially related to lifestyle diseases).		free text (brief summary of aims)
13	**“Lifestyle disease” and/or "civilisation diseases" definition**	Definition or description of “lifestyle disease” and/or "civilisation disease" (and their synonymes like "life-style disease" or "civilisational disease" etc.) used in the study, including differences between both terms, if any mentioned.		free text
14	**Risk factors/determinants**	Key underlying causes, risk factors or determinants of lifestyle diseases discussed.		free text (e.g., diet; physical inactivity)
15	**Identified conditions**	Specific health conditions identified or discussed as lifestyle diseases.		free text (e.g., obesity; diabetes)
16	**Emerging factors/trends**	Emerging lifestyle-related factors or trends contributing to new or evolving lifestyle diseases.		free text (e.g., e-cigarettes; increased screen time)
17	**Preventive measures**	Preventive measures, interventions, or lifestyle recommendations mentioned for preventing or managing lifestyle diseases.		free text (e.g., exercise; dietary changes; policies)
18	**Research gaps**	Research gaps, unmet needs or future research directions noted by the authors in the lifestyle-disease domain.		free text (any noted gaps or suggestions)
